# Investigations of the effect of the amount of biochar on soil porosity and aggregation and crop yields on fertilized black soil in northern China

**DOI:** 10.1371/journal.pone.0238883

**Published:** 2020-11-17

**Authors:** Liang Jin, Dan Wei, Dawei Yin, Baoku Zhou, JianLi Ding, Wei Wang, Jiuming Zhang, Shaojun Qiu, Chengjun Zhang, Yan Li, Zhizhuang An, Jialin Gu, Lei Wang

**Affiliations:** 1 Plant Nutrition and Resources Institute, Beijing Academy of Agriculture and Forestry Sciences, Beijing, China; 2 The Key Laboratory of Soil Environment and Plant Nutrition of Heilongjiang Province, Heilongjiang Academy of Agriculture and Science, Soil Fertilizer and Environment Resource Institute, Harbin, China; 3 College of Agricultural Science and Technology, Heilongjiang Bayi Agricultural University, Daqing, Heilongjiang, China; 4 Key Laboratory of Plant Nutrition and Fertilizers, Ministry of Agriculture and Rural Affairs/ Institute of Agricultural Resources and Regional Planning, Chinese Academy of Agricultural Sciences, Beijing, China; 5 College of Resources and Environment, Northeast Agricultural University, Harbin, China; Tennessee State University, UNITED STATES

## Abstract

The combination of chemical fertilizer and biochar is regarded as a useful soil supplement for improving the properties of soil and crop yields, and this study describes how the biochar of maize straw can be used to improve the quality of the degraded black soil. This has been achieved by examining the effects of combining different amounts of biochar with chemical fertilizer on the porosities and aggregate formation of soil and exploring how these changes positively impact on crop yields. A field trial design combining different amounts of maize straw biochar [0 (NPK), 15.75 (BC1), 31.5 (BC2), and 47.25 t ha^−1^ (BC3)] with a chemical fertilizer (NPK) has been used to investigate changes in the formation of soil aggregate, clay content, soil organic carbon (SOC), and crop yields in Chinese black soil over a three year period from 2013 to 2015. The results of this study show that the addition of fertilizer and biochar in 2013 to black soil results in an increased soybean and maize yields from 2013 to 2015 for all the treatments, with BC1/BC2 affording improved crop yields in 2015, while BC3 gave a lower soybean yield in 2015. Total porosities and pore volumes were increased for BC1 and BC2 treatments but relatively decreased for BC3, which could be attributed to increased soil capillary caused by the presence of higher numbers of fine soil particles. The addition of biochar had a positive influence on the numbers and mean weight diameters (MWD) of soil macroaggregates (>0.25 mm) that were present, with the ratio of SOC to TN in soil macroaggregates found to be greater than in the microaggregates. The most significant amount of carbon present in macroaggregates (>2 mm and 0.25–2 mm) was observed when BC2 was applied as a soil additive. Increasing the levels of maze straw biochar to 47.25 t ha^−1^ led to an increase in the total organic carbon of soil, however, the overall amount of macroaggregates and MWD were decreased, which is possibly due to localized changes in microbial habitat. The supplementation of biochar increased in the amount of aromatic C present (most significant effect observed for BC2), with the ratio of aliphatic C to aromatic C found to be enhanced due to a relative reduction in the aliphatic C content with >2 mm particle fraction. These changes in organic carbon content and soil stability were analyzed using univariate quadratic equations to explain the relationship between the type of functional groups (polysaccharide C, aliphatic C, aromatic C, aliphatic C/aromatic C) present in the soil aggregates and their MWDs, which were found to vary significantly. Overall, the results of this study indicate that the use of controlled amounts of maize-straw biochar in black soil is beneficial for improving crop yields and levels of soil aggregation, however, the use of excessive amounts of biochar results in unfavorable aggregate formation which negatively impacts the yields of crop growth. The data produced suggest that aromatic C content can be used as a single independent variable to characterize the stability of soil aggregate when biochar/fertilizer mixtures are used as soil additives to boost growth yields. Analysis of soil and crop performance in black soil revealed that the application of maize-straw biochar at a rate of 15.75 and 31.5 t ha^−1^ had positive effects on crop yields, soil aggregation and accumulation of aromatic C in the aggregate fractions when a soybean-maize rotation system was followed over three years.

## Introduction

As the world’s largest developing country, China currently has to feed nearly 20% of the world’s population, using only 7% of the globes cultivatable land. China’s population is estimated to reach a peak of approximately 1.445 billion by 2030 [1] when it has been estimated that 550 billion kg of grain will be required to feed the Chinese population. This will require the availability of at least 122 million hectares of cultivatable land and 103 million hectares of permanent basic farmland to ensure that these crop targets are reached [2]. Heilongjiang is one of the major grain-producing bases in China, with long-term excess agricultural production has led to a deterioration in the physical and chemical properties of its soils. Weak soil structures and ongoing environmental damage to its aggregates and porosities are generally thought to be responsible for the decline in soil quality in degraded black soil areas used for agricultural production [3]. Application of a combination of external organic matter and chemical fertilizers has been recommended as one of the most sustainable and environmentally friendly methods to increase soil fertilities and crop yields of these types of soils [4].

The application of maize straw to the soil is beneficial for the formation of soil aggregate structures, whose application can be used in conjunction with fertilizers and water conservation practices to improve the physical, chemical properties and productivity of agricultural soils. In 2015, about 159 million tons of straw were collected in Northeast China, which accounted for around 19% of the total straw output of the whole of China. 44.97 million tons of this corn straw was obtained via a collection of Heilongjiang farmland waste [5, 6]. Much of this corn straw is used for the preparation of biochar, which enables the volume of the corn straw to be reduced to around a 1/3 of its original biomass after carbonization [7]. Biochar is a porous carbon-rich organic matter formed from pyrolysis of agricultural and forestry wastes, which has a highly aromatic ring molecular structure containing multiple functional groups. It is often used to improve the physical and chemical properties of soil [8, 9] resulting in improvements in carbon sequestration, reductions in global emissions [10] and better adsorption of organic and inorganic pollutants [11]. This can lead to significant increases in crop yields and more efficient fertilizer utilization, For example, Hunter et al. (*2017*) have shown that application of masticated cherry wood biochar (2% by weight, produced at 375°C) to soil containing lettuce seedlings resulted in improved crop growth yields [12]. Jin et al. (*2019*) investigated the effects of biochar combined with nitrogen fertilizer on soil pH and rapeseed/sweet potato yields in a five-year field trial, reporting that use of 11.4 t.ha^−1^ of biochar was the optimum level for increasing crop yields [13].

Although many reports of biochar addition resulting in improved crop yields have been reported, some studies have described contradictory negative results for specific soil conditions [14], with improvements in soil quality affording no improvements in crop growth yields. Jeffery et al. *(2017)* reported that biochar addition is most likely to benefit agriculture in low-nutrient, acidic soils that are present in the tropics [15]. For example, Haider et al. *(2017)* reported that although biochar (0, 15, 30 Mg ha^−1^) reduced nitrate leaching and improved soil moisture content in temperate sandy soils over a four year period there was no corresponding increase in maize and summer barley crop yields, which was attributed to soil N deficiency and/or drought stress [16]. Wang et al. (*2019*) showed that application rates of BC20 t ha^−1^ 30 t ha^−1^ and N30 t ha^−1^ = significantly increased plant productivity by 48.3 and 15.7%, respectively, however, no significant increase in yield was observed for lower levels between 20 and 30 t.ha^−1^ [17]. Higher biochar levels can have a negative effect on plant growth or soil properties, with Niu et al. (*2017*) reporting that whilst N fertilization with biochar application at 3 t.ha^−1^ resulted in reduced yield-scaled N_2_O emissions from maize fields, reductions in wheat yields were observed when biochar levels of 12 t ha^−1^ were used in soils of the North China Plain [18]. Dong et al. (*2017*) found that no chemical structural differences were present when fresh and aged biochar was employed as an additive, indicating that the amounts of biochar (30, 60 and 90 Mg ha^−1^) used in this study were optimal in field conditions where soils had been subjected wetting/drying, freezing/thawing, crop cycling and tillage over a five year period [19]. Madari et al. (*2017*) evaluated the effect of varying the amount of wood biochar and synthetic fertilizer (0, 2, 4, 8 and 16 Mg ha^−1^) over a five year period, finding that SOC levels were not increased until the third year, biochar application had no effect on PAW, whilst BD levels were only increased in year 5 [20]. Sun et al.(*2019*) evaluated the effects of applying varying amounts of biochar (i.e., 0, 5, 10, 20, 30, 40 and 50 t ha^−1^) on wheat plant growth in saline soils, finding that excess biochar (more than 30 t ha^−1^) had a negative effect on both the NUE (nitrogen use efficiency) and GY (grain yield) content of wheat [21]. The majority of biochar research into crop growth yields that have been conducted to date that has been carried out in red, saline and sandy soils in temperate regions, with few studies having been carried out exploring the effects of using biochar in degraded boreal environments. Consequently, this study aimed to investigate what effects adding maize biochar/fertilizer mixtures to degraded black soil would have on soil quality and crop yields of farmland in Northeast China [22].

## Materials and methods

### The site, soil, and biochar properties

A long-term biochar field experiment was carried out in Minzhu town in the Daowai district of Haerbin city, China (E126°51´05", N45°50´3"). Meteorological data indicated that the long-term average annual rainfall in this region ranged from 486.4 to 543.6 mm, with 80% of precipitation occurring from June to September in soil classified as Chernozem. Average elevation levels were 138 m above mean sea level with a depth to the groundwater table of 80 m. The annual average wind speed was 4.1 m.s^−1^, with a maximum recorded wind speed of 18.9 m.s^−1^.

The surface soil (0–30 cm) was found to contain 163.3 g.kg^−1^ of available nitrogen, 20.6 g.kg^−1^ of available phosphorus, 187.9 g.kg^−1^ of available potassium and 29.8 g.kg^−1^ of organic matter. The soil had a pH of 6.74 and composition of 56.3% silt, 21.9% clay, and 21.8% sand, with an overall bulk density of 1.31 g.cm^−3^.

The corn straw biochar used in this study contained a mixture of particle sizes that were classified as <0.1 mm (15%), 0.1–2 mm (60%) and >2 mm (25%), that gave a pH of 8.69 with a SOC of 598 g.kg^−1^. Elemental analysis revealed that the biochar contained 16.6 g.kg^−1^ of O, 7.9 g.kg^−1^ of N, 1.3 g.kg^−1^ of P, 17.0 g.kg^−1^ of K, 60.0 g.kg^−1^ of Si, 2.0 g.kg^−1^ of Mg and 3.0 g.kg^−1^ of Ca. The analysis results of the physical properties of biochar show that the average porosity is 11.9nm and the average particle size is μm.

### Soil treatments

Field experiments were carried out using soybean as a crop in 2013 and 2014, with soil treatments carried out in triplicate via application of four biochar/fertilizer formulations, namely: NPK; NPK+15.75 t.ha^−1^ of biochar (BC1); NPK+31.50 t.ha^−1^ of biochar (BC2); and NPK+47.25 t.ha^−1^ of biochar (BC3). The proportion of BC1, BC2, and BC3 is 1: 2: 3. Subsurface fertilizer comprised of 47 kg N ha^−1^, 78 kg P_2_O_5_ ha^−1^ and 68 kg K_2_O ha^−1^ was applied to all of the soybean crops cultivated. Spring soybean cultivar HEINONG 58 was sown during May 2013/2015 and harvested in October 2013/2015, while Spring maize cultivar LONGDAN 42 was sown on May 10 and harvested on October 5 in 2014. All the chemical fertilizer (NPK) preparations were applied to the soil before the soybean plants were sown. The basal fertilizer (NPK) used for maize consisted of 50% urea + total phosphorus and potassium, with a topdressing containing 50% urea used at the jointing stage. The biochar used as a soil amendment was added to furrows dug beside the planting ridges, and then thoroughly mixed into the soil using a ploughing machine set to plow to a depth of over 20 cm. Biochar (supplied by Liaoning Biochar Engineering Technology Center) was prepared through pyrolysis of maize straw at 500°C.

For the determination of soil physical and chemical properties, N and P were determined by chloroform fumigation extraction method [23], K by molybdenum antimony resistance colorimetry [24], soil organic carbon by organic carbon analyzer [25], and soil pH by acidity meter [26].

Physical and chemical properties of topsoil and composition of biochar are shown in Tables 1 and 2.

**Table 1:** Chemical and physical properties of soil top layers in experimental plots (American system).

Depth	Mechanical Composition (%America)			Texture	Available N	Available P	Available k	SOM	pH	Bulk Density
(cm)	Sand	Silt	Clay		(mg/kg)	(mg/kg)	(mg/kg)	(g/kg)		(g/cm^3^)
0–30	21.8	56.3	21.9	silty clay loam	163.3	20.61	187.92	29.87	6.74	1.31

**Table 2:** Biochar composition.

Particle Components %											
SOC (g/kg)	O (g/kg)	N (g/kg)	P (g/kg)	K (g/kg)	Si (g/kg)	Mg (g/kg)	Ca (g/kg)	pH	<0.1 mm	0.1–2 mm	>2 mm
598	166	7.85	1.327	17.0	60	2	3	8.69	15.0	60.2	24.8

^a^SOC indicates soil organic carbon.

^b^O suggests oxygen.

### Measurements

#### Determination of pore-size-distributions in soil

Soil pore-size distributions (PSD) were determined using Mercury intrusion porosimetry (MIP) (Autopore IV 9500, Micromeritics Inc. USA), with intruded mercury volumes measured as the pressure of mercury was increased from 0.0036 to 310 MPa. These MIP tests afforded cumulative intruded mercury volumes that were a function of equivalent pore radii (EPR), with results plotted using two graphical forms: cumulative pore volume vs. logarithmically EPR; and differential PSD vs. logarithmic differentiation dV / d log r. The pore radii range obtained from the cumulative and differential curve analyses were found to be 0.003 lm and 360 lm, respectively, with this wide range allowing for detection of diverse soil pore classes from the PSD curves. Pores were classified into five size classes according to their equivalent pore diameters (EPD): macropores (> 75 lm), mesopores (30–75 lm), micropores (5–30 lm), ultramicropores (0.1–5 lm) and crytopores (0.1–0.007 lm).

#### Soil composition

Whole-soil samples were separated into five particle-size fractions: clay (>2 μm), fine silt (2–6.3 μm), medium silt (6.3–20 μm), coarse silt (20–63 μm) and sand (63–2000 μm). A 100-g soil sample was weighed into a beaker containing 500 mL deionized water employs an ultrasonic vibrator (Sonics Vibracell 600 with Sonotrode CV 26; Sonics & Materials, Newton, CT), with the soil slurry then sonicated at 60 J mL^−1^ at 20°C to agitate the soil sample, while preserving particulate organic matter from disruption [27]. The sand fraction was then separated by wet sieving the soil through a 63-μm mesh screen, with further deionized H_2_O then added to the residual soil slurry to give soil to solution ratio of 1:10 that was subsequently sonicated at 440 J mL^−1^. Gravity sedimentation in water was then used to separate the resultant suspension (according to Stoke’s law) into a range of particle-size fractions, with this sedimentation process repeated 12 times for each sample. Clay fractions were centrifuged at 12000 rpm and then freeze-dried to obtain a powdery residue, with all other fractions air-dried at 35°C after their clear supernatants had been decanted off. The soil clay values that were obtained in these experiments are listed in Fig 2.

#### Soil aggregates and their corresponding stability parameters

Determination of the size distribution of wet-sieved aggregates was performed using a rototap machine containing a nest of eight 100 mm diameter sieves with mesh openings of 10, 7, 5, 2, 1, 0.5, and 0.25 mm, respectively. The weight of soil retained on each sieve was measured after dry sieving 0.5 kg of soil for 2 min. 50 g of the soil aggregate that was retained on the 100 mm sieve was then used for wet-sieving analysis which was carried out mechanically using Yoder’s apparatus containing a nest of sieves with mesh sizes of 2, 1, 0.5, 0.25, and 0.106 mm, respectively. Wet sieving of soil samples was continued for 20 min using 40 oscillations per min, with retained aggregates on each sieve then transferred to a set of pre-weighed beakers that were then oven-dried at 105°C for 24 h, before being weighed. Mean weight diameters (MWD) and geometric mean diameters (GMD) were calculated as indexes of aggregation using eqs 1 and 2:

$$MWD=\frac{\sum_{i=1}^{n}\left(\bar{d_{i}}W_{i}\right)}{\sum_{i=1}^{n}W_{i}}$$ (1)

$$GMD=EXP[\frac{\sum_{i=1}^{n}W_{i}\text{ln}(\bar{d_{i}})}{\sum_{i=1}^{n}W_{i}}]$$ (2)

Where *d*_*i*_ is the mean diameter of the class (mm) and Wi is the proportion of aggregates retained by the sieves relative to the whole sample. According to Peilin Yang et al. [28], the fractal dimension of the sample may then be determined using eq 3:

$$\frac{M(r<\bar{X_{i}})}{M_{T}}={\left(\frac{\bar{X_{i}}}{X_{\text{max}}}\right)}^{3-D}$$ (3)

Where $M(r<\bar{X_{i}})$ is the cumulative mass of objects or fragments of the i^th^ size,$r<\bar{X_{i}}$; MT is the total mass of particles; $\bar{X_{i}}$ is the mean particle diameter (mm) of the i^th^ size class; *X*_max_ is the mean diameter of the largest particles.

#### Fourier transform infrared spectroscopic analysis

Three containers containing soil slurries were stored at room temperature (20°C) for six hours to allow them to equilibrate. The contents of each pack were then shaken before a 0.5 ml sample was pipetted out into an Attenuated Total reflectance (ATR) cell. Sample spectra were then acquired using a PerkinElmer Frontier FTIR spectrometer (PerkinElmer, MA, USA) over a spectral range of 4000–600 cm^−1^ (with a resolution of 4 cm^−1^), using 16 scans to acquire each spectrum. The background spectrum was scanned at the beginning of the measurement using a blank Diamond ATR cell. The spectra of three portions of each sample were acquired by refilling the ATR cell three times. Spectra of dried sediments corresponding to days 0 and 28 of storage for SM and WM were also acquired to estimate the overall composition of the sediment produced.

#### Statistical analysis

All data were analyzed using the Microsoft Excel (2003) software package, with the significance of differences between different treatments and sampling dates determined by ANOVA analysis using the SPSS software package (SPSS Inc., 2003). Differences between values were statistically significant for p<0.05, with coefficients of determination (R^2^) of nonlinear regression used to identify the best fit for the water retention model in soil.

## Results

### Crop yields from 2013 to 2015

**Fig 1:**
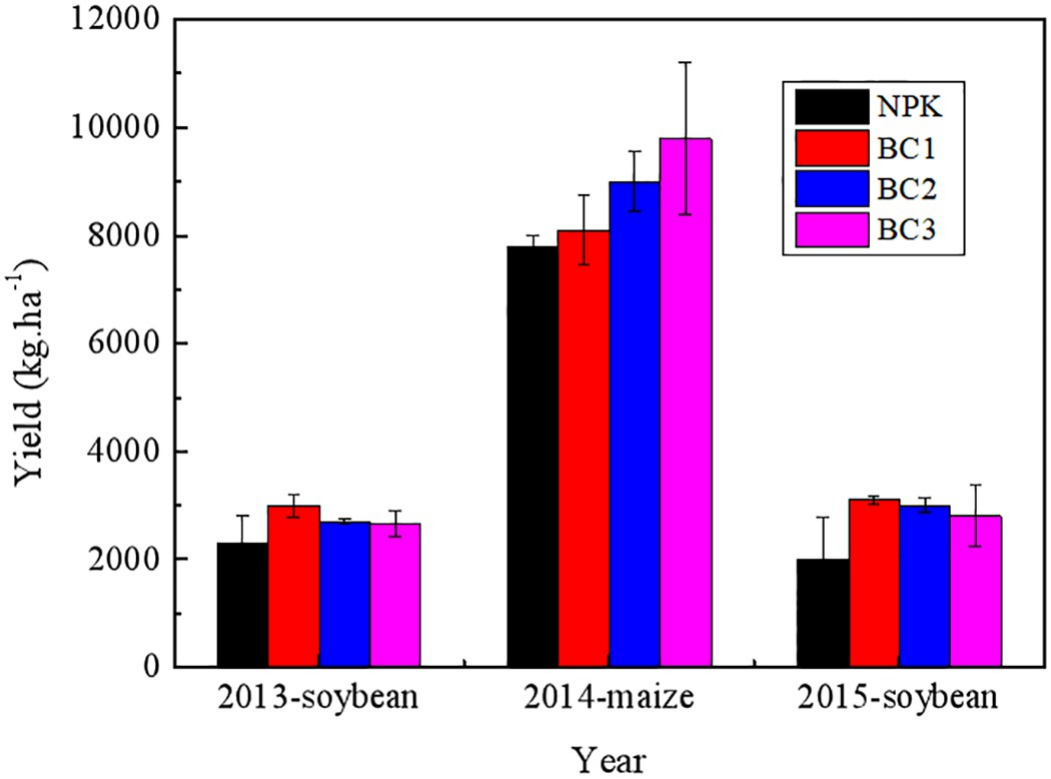
Crop yields from 2013–2015 for the addition of different amounts of biochar.

Fig 1 show the crop yields with different amounts of biochar from 2013 to 2015.

The effect of adding chemical fertilizer combined with different amounts of maize straw biochar application rates on crop yields in black soil was investigated over a continuous three-year period (Fig 1). In 2013, the oil yield from soybean treated with the addition of NPK/biochar was significantly increased from 13.8 to 16.9%, relative to NPK alone. A similar trend for maize production was found in 2014, with crop yields enhanced from 5.2% to 20.3%, for increasing amounts of biochar. In 2015, soybean yields for the highest levels of biochar treatment of 47.5 t ha^−1^ (BC3) were found to be unexpectedly reduced relative to the lower biochar levels of BC1 and BC2.

### Total porosity and pore distribution levels of soil after the addition of biochar over three years

The total porosity and pore-size distribution of the biochar-treated soil are shown in Table 3.

**Table 3:** Total porosity and pore-size distribution of biochar-treated soils after a 3-year treatment period.

Treatment	Total porosity %	Pore distribution/ cm^3^g^−1^						
		Total pore volume /cm^3^g^−1^	>75 μm	30–75 μm	5–30 μm	0.1–5 μm	0.1–0.01 μm	<0.01 μm
**NPK**	48.01^d^	0.2552^d^	0.0966^b^	0.0243^b^	0.0361^c^	0.0602^d^	0.0258^c^	0.0122^ab^
**BC1**	51.91^a^	0.2735^a^	0.0908^d^	0.0266^c^	0.0398^a^	0.0703^a^	0.0329^a^	0.0131^a^
**BC2**	53.42^b^	0.2771^b^	0.0879^c^	0.0217^a^	0.0440^b^	0.0787^b^	0.0327^a^	0.0121^ab^
**BC3**	49.33^c^	0.2628^c^	0.0989^a^	0.0210^d^	0.0403^b^	0.0615^c^	0.0289^b^	0.0122^ab^

Addition of biochar exerted a significant effect on the total porosity, and total pore volume of soils, with the total porosity increased by 8.1% (BC1), 11.3% (BC2) and 2.7% (BC3), respectively, with changes in pore size (measured using mercury porosimetry) distributed throughout all six fractions (>75, 30–75, 5–30, 0.1–5, 0.1–0.01 and <0.01μm) analyzed. After three years, biochar application was found to have increased the numbers of macro- (>30μm), medium- (2~30μm) and micro- pores (<2μm) most significantly, with the little overall impact on the number of micropores (<0.01μm) present. Compared with the NPK control, the total pore volume of the soil was increased by 6.6% for BC1, by 8.8% for BC2 and by 2.4% for BC3, respectively, with any limitations of biochar (for 34.5 t ha^−1^) supplementation on total pore volume caused by a reduction in the numbers of 5–30 and 0.1–5μm pores present.

### Composition of soil aggregate and their SOC concentrations

The results of aggregate composition and stability index of black soil treated with different biochar are shown in Table 4.

**Table 4:** Composition and stability indexes of black soil aggregates for different biochar treatments.

Treatments	Aggregate composition %				Macro-aggregates (>0.25mm)	MWD* /mm
	>2 mm	2–0.25 mm	0.25–0.106 mm	<0.106 mm		
**NPK**	12.76d	52.53a	21.94a	12.77b	65.29d	0.50d
**BC1**	22.09b	50.16b	14.72c	13.03b	72.25b	0.66b
**BC2**	28.04a	47.55d	14.57c	9.84c	75.59a	0.77a
**BC3**	19.62c	49.02c	16.03b	15.33a	68.64c	0.61c

^c^MWD: Mean weight diameter

Distribution of the water-stable aggregate fractions and their relative structural stabilities were changed by biochar addition (Table 4), with macroaggregate structures found to predominate after three years. Compared with the control, significant increments were found in the 0.25–0.5mm fraction, with increases of 2.6% for BC1, 16.9% for BC2 and 23.3% for BC3), respectively. The amount of >2 mm fraction present in the biochar treated soil samples were found to be reduced relative to their 0.25–0.5mm fractions (correlation coefficient of -0.87 (P<0.05, N = 9)), indicating that their structural stabilities had been improved significantly (highest MWD = 0.77 for BC2).

### Regression analysis of clay concentrations and MWD values of soil aggregates

**Fig 2:**
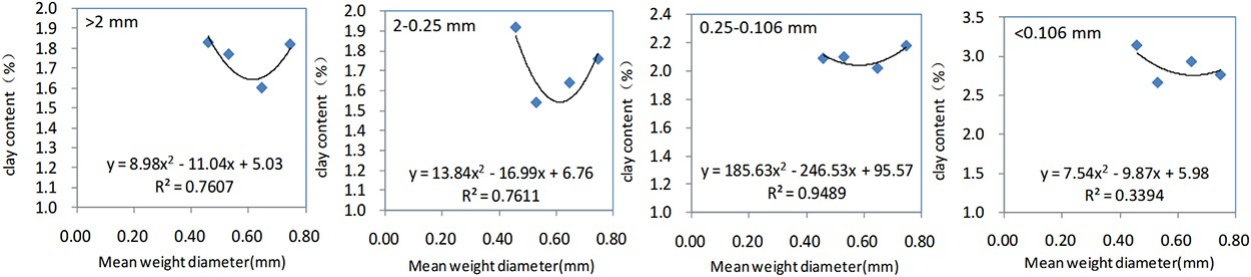
Regression analysis of clay concentrations and MWD values of soil aggregates for different amounts of biochar.

The regression analysis results of clay concentrations and MWD values of soil aggregates for different amounts of biochar are shown in Fig 2.

Clay is an essential component of soil that regulates the degradation and transformation of organic carbon through the formation of aggregates [29] that are known to affect the ion adsorption and infiltration properties of soil [30]. Univariant quadratic regression equations were used to calculate the relationship between clay concentration in aggregate fractions and their MWD values. The coefficients of regression equations for particle sizes of 0.25–0.106 mm was found to be 0.94, affording a larger value than was obtained for the >2, 2–0.25 and <0.106 mm particles. The results of total organic carbon content in soil aggregates under different biochar additions are shown in Table 5.

**Table 5:** Total organic carbon in soil aggregates for the addition of different amounts of biochar.

		SOC concentration				SOC/TN			
Treatment	TOC	>2 mm	2–0.25 mm	0.25–0.106 mm	<0.106 mm	>2 mm	2–0.25 mm	0.25–0.106 mm	<0.106 mm
		g kg^−1^	g kg^−1^	g kg^−1^	g kg^−1^				
**NPK**	17.78d	15.24 c	16.62 c	15.32 b	12.50 b	6.33	7.63	6.77	6.04
**BC1**	19.49c	17.28 b	17.92 c	15.12 b	13.31 a	6.53	7.51	5.64	5.66
**BC2**	20.67b	25.46 a	24.92 a	18.44 a	14.09 a	10.38	10.03	7.15	5.96
**BC3**	21.37a	17.94 b	22.64 b	18.69 a	15.51 a	8.41	9.87	7.93	5.91

^d^TOC represents total organic carbon.

^e^TN is the total nitrogen.

^f^SOC suggests soil organic carbon.

Addition of biochar had a significant influence on SOC concentrations in aggregate fractions (Table 4), with SOC concentrations enhanced from 12.5 to 25.5 g kg^−1^ for increasing amounts of biochar. Compared with the NPK control, higher SOC concentrations resulted in an increase in the number of >2mm aggregates (from 13.4% to 67.0%) and 2–0.25 mm aggregates (from 7.8 to 49.9%). Increasing biochar content resulted in SOC concentrations in aggregates with a size of >2mm and 2–0.25 mm being increased for BC1and BC2 treatments; however, levels were reduced for BC3. The ratios of SOC/TN distributed throughout the aggregate fractions ranged from 5.64–10.38, with the largest ratio found in the >2 mm fraction after BC2 treatment, and the lowest ratio observed in the 0.25–0.106 mm fraction after BC1 treatment. The ratio changes in macroaggregate fractions (>2 mm and 2–0.25 mm) were found to range from 6.33 to 10.38 (average of 7.91) and from 7.63 to 10.03 (average of 7.91), with the ratio changes in micro-aggregates (0.25–0.106 mm and <0.106 mm) ranging from 5.64 to 7.93 (average of 6.87) and 5.66 to 6.04(average of 5.89 in average).

### Characterization and semi-quantitative analysis of functional groups present in SOC in soil samples

#### Fourier transform-infrared spectroscopic analysis of soil aggregates

**Fig 3:**
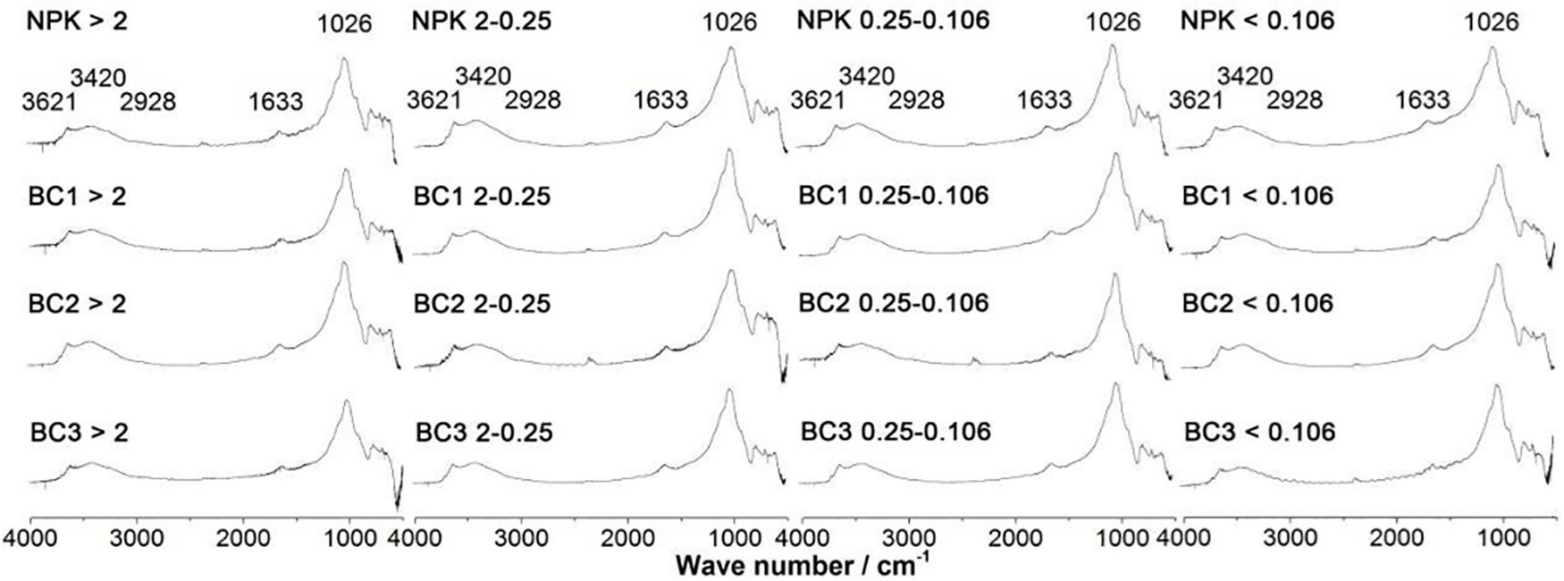
Effect of biochar content on organic carbon functional groups absorption spectra of soil samples.

The effect of biochar content on the absorption spectra of organic carbon functional groups of soil samples is shown in Fig 3.

**Fig 4:**
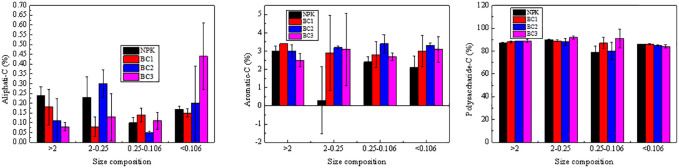
Composition of organic carbon functional groups present in soil aggregates.

Fig 3 shows the organic carbon functional groups absorption spectrum for soil aggregates with various biochar content, with absorption bands observed for polysaccharide-C (1026 cm^−1^), aromatic–C (1633 cm^−1^) and aliphatic–C (1633 cm^−1^). Absorption intensities for aliphatic–C content were not significantly changed for increasing amounts of biochar application. Absorption intensities for aromatic–C were all weakened when compared to NPK (control) treatment, whereas BC1 and BC2 treatment resulted in the aromatic–C content of all aggregates being increased, with BC2 giving the highest values. Compared with NPK, the absorption intensities of polysaccharide-C in biochar treated samples all showed a decline for aggregate sizes of 2–0.25mm and 0.25–0.106mm. The composition of organic carbon functional groups in soil aggregates is shown in Fig 4.

Overall, the content of functional groups present soil aggregates were in the following order: polysaccharide-C > aromatic–C > aliphatic–C. Aromatic–C content in aggregates with a size of >2 mm were maximal for BC2 treatments, with the aromatic–C content in aggregates with a size of 2–0.25mm and 0.25–0.106 mm increased, and their polysaccharide-C content decreased. Increasing biochar levels resulted in greater aliphatic–C content in macroaggregates (>2mm and 2–0.25mm), affording a 2.1% increase for BC1, 4.6% for BC2 and 4.2% BC3, which corresponds to a 38.0% increase, respectively. Aromatic–C was found to accumulate throughout the three fraction sizes (>2mm, 2–0.25mm and <0.106mm), with the BC2 application increasing by 81%, 42% and 8% for each aggregate size, respectively.

#### Relationship between organic carbon functional groups and aggregate stability of soil samples

**Fig 5:**
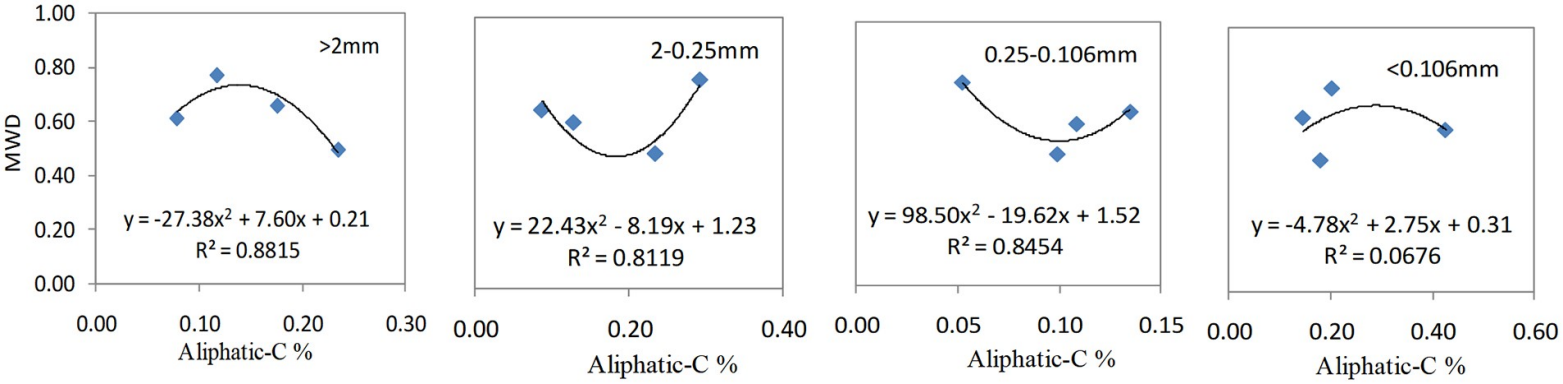
Regression models of aliphatic–C and soil aggregate stabilities for different particle sizes.

**Fig 6:**
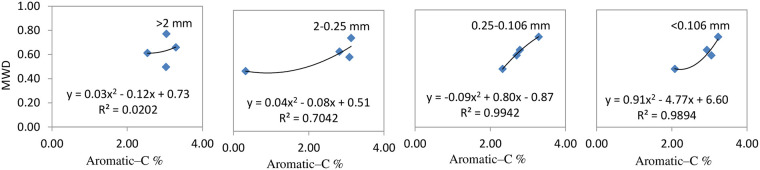
Regression models of aromatic–C and soil aggregate stabilities for different particle sizes.

**Fig 7:**
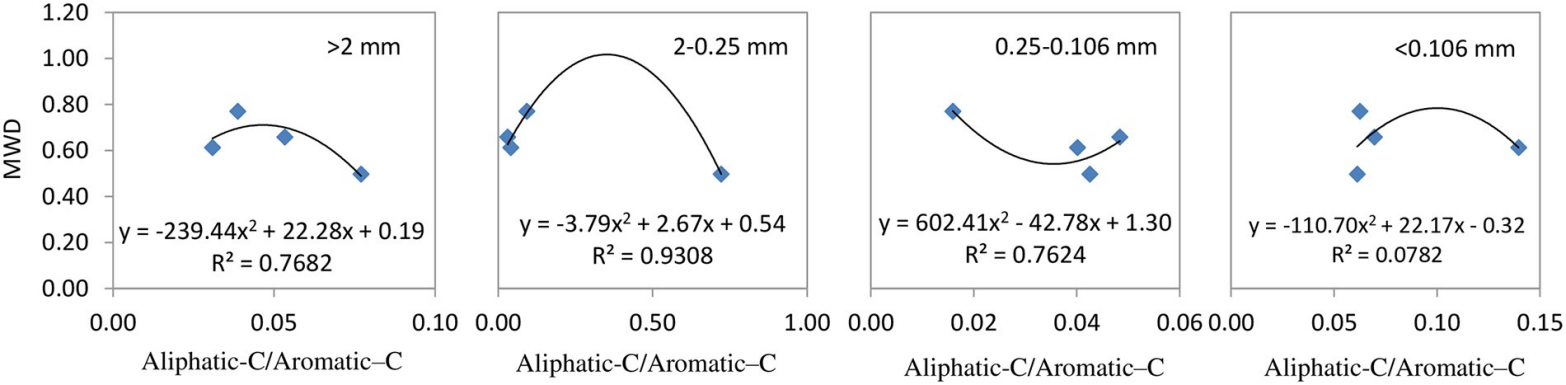
Regression models of aliphatic–C/aromatic–C content and soil aggregate stabilities for different particle sizes.

Figs 5 to 7 reveal that the correlation between SOC functional group indexes and soil aggregate stabilities (simulated by quadratic equations) was greater than for other mathematical analyses (regression equations, logarithmic equations, quadratic polynomial equations, and exponential regression equations) carried out.

The regression model used for analysis of particle sizes of >2 mm were described as Y = -27.38X^2^ + 7.60X + 0.21, Y: MWD, X: aliphatic–C; particle sizes of 2–0.25 mm were described as Y = -3.79X^2^ + 2.67 X + 0.54, X: aliphatic–C / aromatic–C; particle sizes of 0.25–0.106 mm fraction were described as Y = -0.09 X^2^ + 0.80X—0.87; particle sizes of <0.106 mm fraction were described as Y = 0.91X ^2^+ 4.77 X + 6.60, X: aromatic–C. The R^2^ coefficient for these analyses was all greater than 0.80, with the aromatic–C content maintaining a positive relationship with MWD values, while the other three indexes exhibited a negative correlation with MWD values.

## Discussion

Increased crop yields were obtained from 2013 to 2015, which demonstrated the overall benefit gained from the addition of maize straw biochar, even though soybean yield using BC3 was reduced in 2015 (Fig 1), which is likely to be due to excess biochar altering soil porosities and changing soil aggregate compositions/stabilities. Biochar is a porous organic medium, so its application significantly increased the total porosity and effective pore size of the soil, with the highest total porosity obtained for the addition of biochar addition at 31.5 t.ha^−1^, which resulted in the numbers of pores with diameters of 5~30 μm, 0.1~5 μm, and 0.1~0.001 μm being increased significantly. However, increasing biochar levels also led to total levels of 5~30μm and 0.1~5μm pores being decreased, which led to reduced water retention in soil and an increase in its wilting water content [31]. This is because increased amounts of biochar in the soil generate higher numbers of fine particles that exhibit greater capillary forces under the external forces of mechanical cultivation and rainfall [32]. Nevertheless, our results clearly demonstrated that addition of biochar to soil significantly enhanced the stability of aggregates by increasing their MWD values (Table 4), which was consistent with previous reports by Zong et al. (*2018*) who demonstrated that application of wastewater sludge biochar (WSB) increased the aggregate stability of two Ultisols with different soil textures [33]. Average MWD values were increased over the range of 0.11 to 0.27mm for biochar-treated soil, with the highest increase of 54.1% found for BC2, which had a more significant structural stability relatively to BC3 treated soil. This is likely to be the negative effect of excess biochar (47.25 t ha^−1^) on microbial biomass and nitrogen mineralization processes (per unit soil volume), with the number of soil macroaggregates presents reduced when biochar levels of greater than 31.5 t ha^−1^ were used. This observation is mainly due to excess biochar hindering soil reaggregation which results in a decrease in available nitrogen sources that increase competition for microbial biomass and leads to an overall decrease in activity. Three years of biochar supplementation resulted in the number of water-stable soil macroaggregates present being much improved, with the SOC content of aggregates with particle sizes of >2 mm and 0.25–2 mm found to be higher than for other particle sizes (Table 5). SOC functions as an essential soil binding agent, whose concentration in soil is known to determine overall mineralization rates [34]. We found that application of biochar as an external organic carbon source changed SOC concentration in soil aggregates over time, with most of this SOC being present in macroaggregates (>2 mm and 2–0.25 mm) that is degraded/recycled at a faster rate than when it is present in microaggregates [35]. SOC levels in aggregates with sizes of 2–0.25 mm were found to account for between 26% and 36% of the content of the soil, meaning that this soil fraction is as a major reservoir of organic carbon in black soil [36]. Soil aggregate stabilities are determined by the concentration of carbon (C) concentration and nitrogen (N) they contain, and their relative ratios [37]. The C/N ratios for two sizes of aggregates in soil containing high levels of C saturation ranged from 6.33 to 10.38 (average of 4.05) and 7.38 to 9.99 (average of 2.61), whilst the ratios of the other two aggregate sizes were found to range from 5.64–7.93(average of 2.29) and from 5.66–6.04 (average of 0.38). Therefore, SOC in macroaggregates is more liable to decompose and be transformed into micro aggregates [38], with SOC concentrations in aggregates with a size of >2mm increased from 15.24 g/kg for NPK to 25.46 g/kg for BC2 treatment, while the SOC content of aggregates with a size of 2–0.25mm was increased by 49.9%. Application of larger amounts of biochar led to the overall SOC concentration in macroaggregates being decreased; however, SOC was still relatively increased in aggregates with sizes of 0.25–0.106 mm and <0.106 mm [39].

These findings can be interpreted using the “macroaggregate organization” soil model, where clusters of atoms containing different SOC functional groups are proposed to influence the physical/chemical properties of soil aggregates. Three years of biochar application resulted in the relative intensities of different types of SOC functional groups in the soil being impacted differently (Fig 3). The absorption intensities measured for polysaccharide C were significantly higher than for aromatic C and aliphatic C, with the BC2 treatment producing higher numbers of particle sizes with diameters of 2–0.25 mm, 0.25–0.106 mm and < 0.106 mm, which was consistent with the higher MWD values produced by BC2. Therefore, supplementation with biochar has a much more significant effect on increasing the macroaggregate content of the soil than the corresponding micro aggregate fraction [40].

Increased biochar application (47.25 t ha^−1^) resulted in the amount of aliphatic carbon present in particles with a size of >2 mm being reduced due to oxidative decomposition; however, the overall aliphatic carbon content of other particle sizes was relatively increased. Treatment with BC3 resulted in the amount of aliphatic C to aromatic C substances being relatively increased (Fig 5), results in soil compactness being changed and its stability reduced. Organic carbon in microaggregates is mainly present as humic substances that are generated by microorganisms, which makes it highly resistant to chemical and biological decomposition [41, 42]. The coefficients of regression models for the aromatic C present in microaggregates (particle sizes of 0.25–0.10 and <0.106mm) to their MWD values were greater than 0.9, which was a higher value than the amounts present in aromatic C and MWD in macroaggregates. Therefore, this means that the aromatic C content present in microaggregates appear to be better at stabilizing soil aggregates/structures than macroaggregates.

## Conclusions

This study reports that the use of relatively high amounts of biochar positively influences the benefits of nitrogen fertilizer on crop yields in black soils over two years (2013–2015), with adverse effects only being observed for soybean yields when BC3 (highest biochar level) was used in 2015. The porosities, aggregation properties, and structural stabilities of the soil were significantly strengthened (as estimated by MWD) when it was supplemented with biochar. This resulted in the numbers of soil macroaggregates and its MWD values being increased when biochar was applied at a rate of 31.25 t.ha^−1^ (BC2), which resulted in the saturation of the SOC content of aggregates with diameters of >2 mm and 0.25–2 mm. However, the use of excess biochar (BC3, 47.25 t.ha^−1^) resulted in a reduction in the number of soil macroaggregates, increased the capillary soil levels, total soil porosity values, and MWD levels. The total N content and the type of SOC functional groups present in the aggregate fractions of black soils were altered in the presence of biochar, with univariant quadratic regression analysis used to estimate the relationship between the functional groups of SOC, aggregate size, and MWD values. These calculations revealed that the aromatic-C content of microaggregates in biochar treated soils could potentially exert a positive influence on their aggregate stabilities. Therefore, these new results provide further evidence of the benefits of using biochar (between 15.75 and 31.5 t.ha^−1^) supplements to improve the performance of soil aggregation and crop yields of farmland which are used for growing soybean/maize crops.

## Supporting information

S1 File (XLSX)
